# Fisetin and polymeric micelles encapsulating fisetin exhibit potent cytotoxic effects towards ovarian cancer cells

**DOI:** 10.1186/s12906-018-2127-7

**Published:** 2018-03-15

**Authors:** Xue Xiao, Juan Zou, Yin Fang, Yibo Meng, Chao Xiao, Jiaxin Fu, Shiyu Liu, Peng Bai, Yuan Yao

**Affiliations:** 1 Department of Obstetrics and Gynecology, West China Second University Hospital, Sichuan University, Chengdu, 610041 People’s Republic of China; 2 Labratory of Genetics, West China Second University Hospital, Sichuan University, Chengdu, 610041 People’s Republic of China; 3 Department of Pathology, West China Second University Hospital, Sichuan University, Chengdu, 610041 People’s Republic of China; 4 Laboratory of stem cell & tissue engineering, West China Second University Hospital, Sichuan University, Chengdu, 610041 People’s Republic of China; 5 Key Laboratory of Obstetric & Gynecologic and Pediatric Diseases and Birth Defects of Ministry of Education, Chengdu, People’s Republic of China; 6 Department of Forensic Biology, West China School of Preclinical and Forensic Medicine, Sichuan University, No.24 South Section 1, Chengdu, Sichuan People’s Republic of China; 7 Department of Obstetritics and Gynaecology, MeiShan City People’s Hospital, Meishan, 620010 People’s Republic of China; 8 Department of Gynaecology and Obstetrics, ZiYang maternal and child health care hospital, West China Second University Union-hospital, Ziyang, People’s Republic of China; 9 Department of Gynaecology and Obstetrics, Zigong maternal and child health care hospital, Zigong, People’s Republic of China; 10 Department of Pathology, The University of Texas M. D. Anderson Cancer Center, Houston, TX USA

**Keywords:** Ovarian cancer, Fisetin, Polymeric micelles encapsulating fisetin, Apoptosis, Antitumor, Mitochondrial pathway

## Abstract

**Background:**

The anti-tumor activities of Natural compounds and their derivatives are of great interest to pharmaceutical industries. Fisetin is one of prospective natural compounds in this regard but unfortunately with poor hydrophilicity.

**Methods:**

The effects of unmodified and modified fisetin in cultured ovarian cancer cells were compared by transmission electronmicroscopy to determine apoptotic bodies, MTT assay to quantitate cell numbers, and fluorescence activated cell sorting analyse of various markers to determine the apoptotic state. In addition, the efficacy of fisetin and fisetin-micelles in vivo was determined by using immunocompromised mice. Apoptosis was measured by established markers using both western blot analysis and immunochemistry. Angiogenesis in a xenograft mouse model carring SKOV3 cells was evaluated by color Doppler ultrasound and immunohistochemistry.

**Result:**

Multiple lines of evidence indicated that fisetin and fisetin micelles induce apoptosis in ovarian cancer cells in a dose-dependent manner. Histological analysis, terminal deoxynucleotidyltransferase-mediated nick-end labeling assay, western blot, immunohistochemical detection and microvessel density detection demonstrated that fisetin and fisetin micelles induced increased tumor apoptosis, proliferation suppression and antiangiogenesis activities.

**Conclusion:**

As far as we know, the present study is the first time to demonstrate the potency of both fisetin and fisetin micelles inducing apoptosis in ovarian cancer cells. Further studies will be needed to validate the therapeutic potential of fisetin and fisetin micelles in ovarian cancer treatment.

## Background

Ovarian cancer has been the leading cause of cancer-related death for women in the past 15 years. In 2017, about 22,440 women have been diagnosed with ovarian cancer and 14,080 have been died from the disease in USA alone. In the absence of an effective early diagnosis, approximately 70% patients are already diagnosed with stage III or IV of ovarian cancer [1]. Taxol/platinum-based chemotherapies were still identified as the most standard and widely used chemotherapy. Other novel, targeted therapies such as PARP, VEGF and proteasome inhibitors, resulting in a dismal 25% five-year OS (overall survival) as well as 70% recurrence [2]. Therefore, identification of novel therapeutic targets and treatment regimens is subjected to intensive research.

Fisetin (3,3′,4′,7-tetrahydroxyflavone) is an attractive candidate for ovarian cancer treatment. Previous studies have showed that fisetin exhibits antitumor effect through various mechanisms [3–7]. Previous studies have found that fisetin has antioxidant [8, 9], anti-inflammatory [10], anti- cell proliferation [11–13], anti-tumor metastasis [14], anti-angiogenesis [15–17], anticancer [16], anti-allergy [18], anti-platelet functions [19]. However, the poor water solubility of fisetin (less than 1 mg/g) retarded the clinical application and commercial production. In current study, we detected the effect of fisetin and fisetin-micelles on ovarian cancer cell line growth, the relative apoptosis pathway and the anti-tumor effect in xenograft mice. The aim is to find new effective and low toxic drugs for treatment of ovarian cancer. Recently, the biodegradable polymeric micelles are gradually gaining attention due to high bioavailability, better selectivity and accurate targeting in the form of drug delivery systems. Polymeric copolymers are self-assembled amphiphilic particles, where the hydrophobic blocks hind the hydrophobic drugs in the central, the hydrophilic blocks can form a shell to contact with aqueous solvent [20]. Theoretically, nanosized particles and increased water solubility possess increased antitumor activity and passive targeting. Moreover, the tension effects in tumor tissues and sustained release mechanism in blood vessels can enlarge their antitumor ability.

In this study, we used monomethyl poly (ethylene glycol)-poly(ε-caprolactone), MPEG-PCL polymeric micelles to modify the physical properties of fisetin. The histological analysis, terminal deoxynucleotidyltransferase-mediated nick-end labeling assay, western blot, immunohistochemical detection and microvessel density detection were used to investigate the antitumor activity of fisetin and fisetin micelles both in vitro and in vivo.

## Methods

### Materials

Fisetin, purification is more than 98%, was provided by the Department of Pharmacology of Sichuan University (Chengdu, Sichuan, China). Monomethyl poly (MPEG, Mn = 2000, Fluka, USA); ε-caprolactone (ε-CL, Alfar Aesar, USA), stannous octoate (Sn(Oct)2, Sigma, USA) MPEG-PCL copolymer was synthesized according to our previous work in State Key Laboratory. The copolymers were characterized by^1^H NMR with molecular weight 3950.SKOV3 human ovarian cancer cell line was provided by Laboratory of Genetics, West China Second University Hospital.RPMI-1640 and fetal bovine serum (FBS) (Gibco BRL, Life Technologies Gaithersburg, MD, USA). Thiazolyl blue (sigma company), VEGF was obtained from (R&D Systems, Minneapolis, MN). VEGF ELISA kit was purchased from R&D Systems (Minneapolis, MN). Growth factor–reduced Matrigel was from BD Biosciences (San Jose, CA, USA). Antibodies against VEGF, VEGFR2, Bcl-2, and Bcl-xL were obtained from Santa Cruz Biotechnology Inc. (Santa Cruz, CA, USA). β-actin antibody was purchased from Sigma Chemical Co. (St. Louis, MO, USA).CD31 antibodies were from EpitomicsInc (Burlingame,CA, USA). Flow cytometry analyzer (Beckman Coulter), Hitachi h-600IV TEM (West China School of Preclinical and Forensic Medicine, Sichuan University). Inverted biological microscope (Chongqing Photoelectric instrument Co, Ltd., XDS-1B), Cell culture box (SANYO, Japan MCO-18 M (UV)), Standard ultra clean bench (SW-CJ-2FD, AIRTECH).

### Cell lines and cell culture

SKOV3 cells, human bronchial cells, vascular smooth muscle cells, OSE, HEC-1A, CASKi and HepG2cells were cultured in RPMI-1640 medium supplemented with 10% fetal bovine serum at 37 °C under a humidified 95%:5% (*v*/v) mixture of air and CO2.When the SKOV3 cells in the culture box covered 70-80% of the bottle, digested with trypsin, collection and centrifugation. Cells in logarithmic growth phase were used in the experiment. All the cells were bought from ATCC (American type culture collection), the partial are primary cells.

### Transmission electron microscopy (TEM)

SKOV3 cells were treated with 100 μmol/L fisetin and fisetin micelles for 24 h. Cells were digested with trypsin after centrifugedand harvested in 2 ml EP tube, centrifuge for 15 min with 2000 rpm, the supernatant was discarded. Cells then fixed in 5% glutaraldehyde and 3% paraformaldehyde, dehydrated in an ascending acetone series, embedded in Epon812 resin, sectioned into ultrathin longitudinal sections, and imaged using a transmission electron microscope (JEOL 1010, Jeol, Tokyo, Japan).

### Fisetin and fisetin micelles treatment and determination of cell survivability

Cells were seeded at a density of 5 × 10^3^ per well in 96-well tissue culture plates after digested with trypsin and diluted with 10% fetus RPMI-1640 culture. Carrier DMSO was used as a negative control. The cultured cells were treated with fisetin and fisetin micelles on different doses(10 μmol/L、30 μmol/L、100 μmol/L and 300 μmol/Lrespectively). Viability of each cell groups was examined by the3-[4,5-dimethylthiazol-2-yl]-2,5-diphenyltetrazolium bromide (MTT; Sigma, USA) assay every 24 h. Time- and dose-dependent curve of fisetin/fisetin micelles-treated cells were generated. The cytotoxic effects of fisetin/fisetin micelles were expressed as the IC50 (half maximal inhibitory concentration) or TGI (total growth inhibiting concentration). Formula lgIC50 = Xm-I(P-(3-Pm-Pn)/4) was used to calculate IC50.

### Flow cytometry analysis for apoptosis

Flow cytometry was used to analyze the loss of membrane symmetry and membrane integrity using FITC Annexin V and propidium iodide (BD ApoAlertAnnexin V-FITC Apoptosis, kit, BD Biosciences), respectively. Briefly, cells were treated with fisetin and fisetin micelles at various concentrations (30 μmol/L、100 μmol/Land 300 μmol/L, respectively) for 48 h. Cells then were digested with non-EDTA trypsin for 3 min and harvested.5 × 10^5^Cells were labeled with propidium iodide (PI) and Annexin V-FITC for 30 min at room temperature. After filtration with a 300 dialyzer (FACSort, Becton Dickinson, San Jose, CA). Data were further analyzed by CellQuest software, version 3.1 (Becton Dickinson) and ModFit LT 3.2 (Verity Software House). Experiments were conducted in triplicate.

### Terminal deoxynucleotidyltransferase-mediated dUTP nick end labeling assay

Cells layers with different dose- and time-treated were fixed with 4% paraformaldehyde solution for 15 min at room temperature, washed with phosphate buffered saline, and then permeated with permeabilization solution (Triton X-100, 0.1% and sodium citrate) at 4 °C for 2 min. Fisetin-treated SKOV3 cells and control were labeled with the TUNEL reaction mixture for 1 h at 37 °C. Images were takenby fluorescence microscopy. (Confocal Scanning Laser Microscopy, Leica TCS4D, Germany).

### Western blotting analysis

SKOV3 cells were treated with fisetin/fisetin micellesat various concentrations(30 μmol/L、100 μmol/L and 300 μmol/L) at 24 h time point. Whole cell lysates were obtained using RIPA Buffer (Sigma, USA). The protein concentrations were determinated using the Bradford assay. In each lane, 25 mg proteins were resolved in 12% sodium dodecyl sulfate polyacrylamide gel electrophoresis (SDS-PAGE). Antibodies (anti-Bcl-2 (1:1000), anti-Bax (1:2000), anti-procaspase-9 (1:1000), anti-caspase-9 (1:1000), anti-procaspase-3 (1:1000), anti-caspase-3 (1:2000) and PARP (1:2000)) incubated the membrane as primary antibodies and HRP-conjugated secondary antibodies were used sequentially. Blots were developed with enhanced chemiluminescence (ECL, Pierce, USA) and exposed to X-ray film.

### The effect of fisetin and fisetin micelles in a xenograft model of ovarian cancer

To investigate if fistetin/fisetin micelles can inhibit tumor growth in vivo, we established a xenograft model of SKOV3 cells in BALB/athymic nude mice SKOV3 cells were digested, harvested and concentrated, then washed and resuspended with new PBS and made the final concentration of cells to be 5 × 10^6^/L. As the model described previously [21, 22], totally 40 4-to 6-week-old female Balb/c nude mice were injected with 5 × 10^6^ SKOV3 cells, led to tumor growth up to 1 week and randomly divided into 8 groups (*n* = 5). Mice bearing tumors were injected with fisetin or fisetin micelles. Mice were injected once every week starting on day 0for 4 times. Control groups were treated with vehicle (DMSO, <0.1%) in saline solution or treated with mPEG-PCL. Gerneral observations of the mice were made twice weekly. Two perpendicular diameters of the xenograft in centimeters were measured to calculate the tumor mass using the formula (a × b^2^)/2. All experiments were conducted according to the guidelines of The Institute for Nutritional Sciences (Shanghai, China).

### Statistical analysis

Statistical significance of differences between groups was performed using the Student’s t-test. Data are presented as mean ± standard deviation (SD). IC50 values were calculated by SPSS (Solutions Statistical Package for the Social Sciences) software version 13.0(SPSS, Inc., China). A value of *p* ≤ 0.05 was considered to be significance. A random number table method was used to make a random grouping, as shown in Table 1.

**Table 1:** Groups of Xenograft model of ovarian cancerand treatment

Groups	Mice number	Drug treatment	Concentration and dosage	frequency	weeks
Group A	5	NS + DMSO	control 0.1 ml	Treatment 5 days and rest 2 days	2 weeks
Group B	5	mPEG-PCL+ DMSO	control 0.1 ml	Treatment 5 days and rest 2 days	2 weeks
Group C	5	fisetin + mPEG-PCL + DMSO	50 mg/kg,0.1 ml	Treatment 5 days and rest 2 days	2 weeks
Group D	5	Fisetin micelles + mPEG-PCL + DMSO	100 mg/kg,0.1 ml	Treatment 5 days and rest 2 days	2 weeks
Group E	5	NS + DMSO	control 0.1 ml	Treatment 5 days and rest 2 days	2 weeks
Group F	5	mPEG-PCL+ DMSO	control 0.1 ml	Treatment 5 days and rest 2 days	2 weeks
Group G	5	fisetin + mPEG-PCL + DMSO	50 mg/kg,0.1 ml	Treatment 5 days and rest 2 days	2 weeks
Group H	5	Fisetin micelles + mPEG-PCL + DMSO	100 mg/kg,0.1 ml	Treatment 5 days and rest 2 days	2 weeks

## Results

### Fisetin and Fisetin micelles treatment inhibits growth of human ovarian cancer cells

**Fig. 1:**
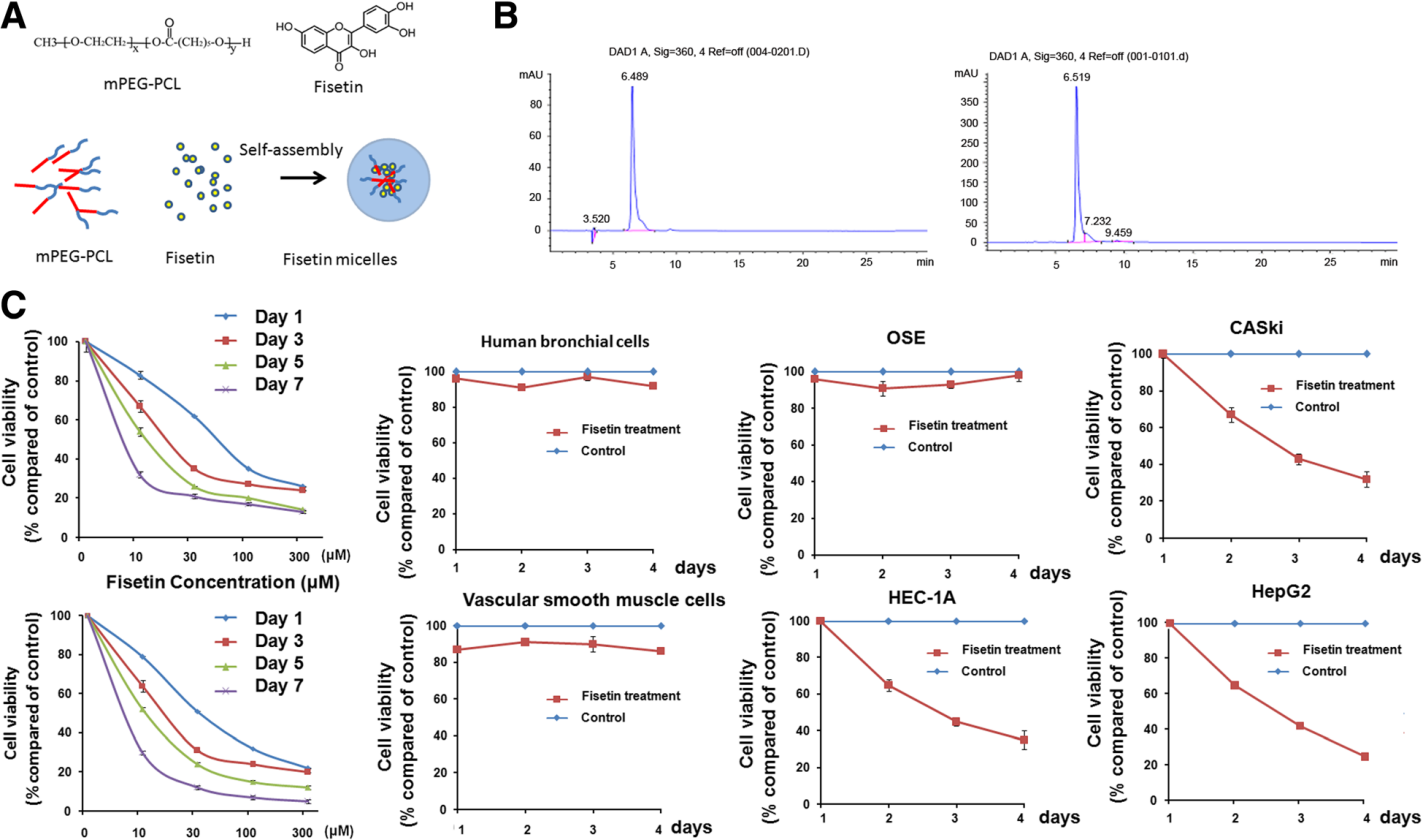
Fisetin and fisetin micelles inhibit tumor cell proliferation. **a** Chemical structure of fisetin, Chemical structure of mPEG-PCL copolymer and preparation scheme of fisetin micelles by the self-assembly method. **b** High performance liquid chromatography of fisetin and fisetin micelles. **c** Various cell lines were treated with30uM fisetin/fisetin micelles for different time periods (1 day to 4 days). Cell viability was detected by MTT assay. Fisetin does not have any inhibit effect on the survival of normal cell lines. Fisetin and fisetin micelles decreae cell viabilities of tumor derived cell lines such as HEC-1A, CASKi, HepG2. Dose-time-effect curves for fisetin and fisetin micelles on the SKOV3 cell line for 1, 3, 5 and 7 days and 10 μM,30 μM, 100 μM, 300 μM, Error bars represent standard error of the mean (SEM) (*n* = 3) *P < 0.05, vs. day 1 group; △*p* < 0.05, vs. control

The chemical structure of fisetin and fisetin micelles is shown in Fig. 1a. The fisetin micelles was prepared according to previous methods, which self-assemble into structures of hydrophilic shells and hydrophobic cores. The average size, polydispersion index, encapsulating efficiency, and drug loading of fisetin micelles were 22.4 ± 3.0 nm, 0.163 ± 0.032, 98.53 ± 0.02%, and 9.88 ± 0.14%, respectively. To investigate the cytotoxic effects of fisetin/fisetin micelles on human ovarian cancer cells, we first treated SKOV3 cells with increasing concentrations of fisetin/fisetin micelles and examined the anti-proliferation effect after24, 48, 72 and 96 h. As shown in Fig. 1, cell numbers were reduced after fisetin/fisetin micelles treatment in a time- and dose-dependent manner in SKOV3 ovarian cancer cells but not in normal cell lines, including human bronchial epithelial cells, human ovarian surface epithelial cells, human vascular smooth muscle cells, etc. At a concentration of 100 μM, the inhibition index of 7-day fisetin-treatment was 83.0%, slightly lower than PSI and etoposide, well known cytotoxic agents. The cell survival curves of the three cancer cell lines (CASki, HEC-1A and HepG2) after fisetin-treatment or fisetin micelles-treatment for 4 days indicated>50% inhibition ratio compared to untreated groups. More specifically, the IC50S for fisetin in tumor derived cell lines CASki, HEC-1A and HepG2 were114.8 μM, 108.9 μM, 126.8μMrespectively. The IC50 and TGI values of fisetin on treated SKOV3 cells were 61.2 μM and 34.8%. The IC50 and TGI values of fisetin micelles on treated SKOV3 cells were 48.2 μM and 48.7%. Furthermore, prolonged exposure time and increased drug dose vastly increased its inhibitory effect on cell number.

### Characterization of Fisetin-induced apoptosis in ovarian cancer cells from transmission electron microscopy results

**Fig. 2:**
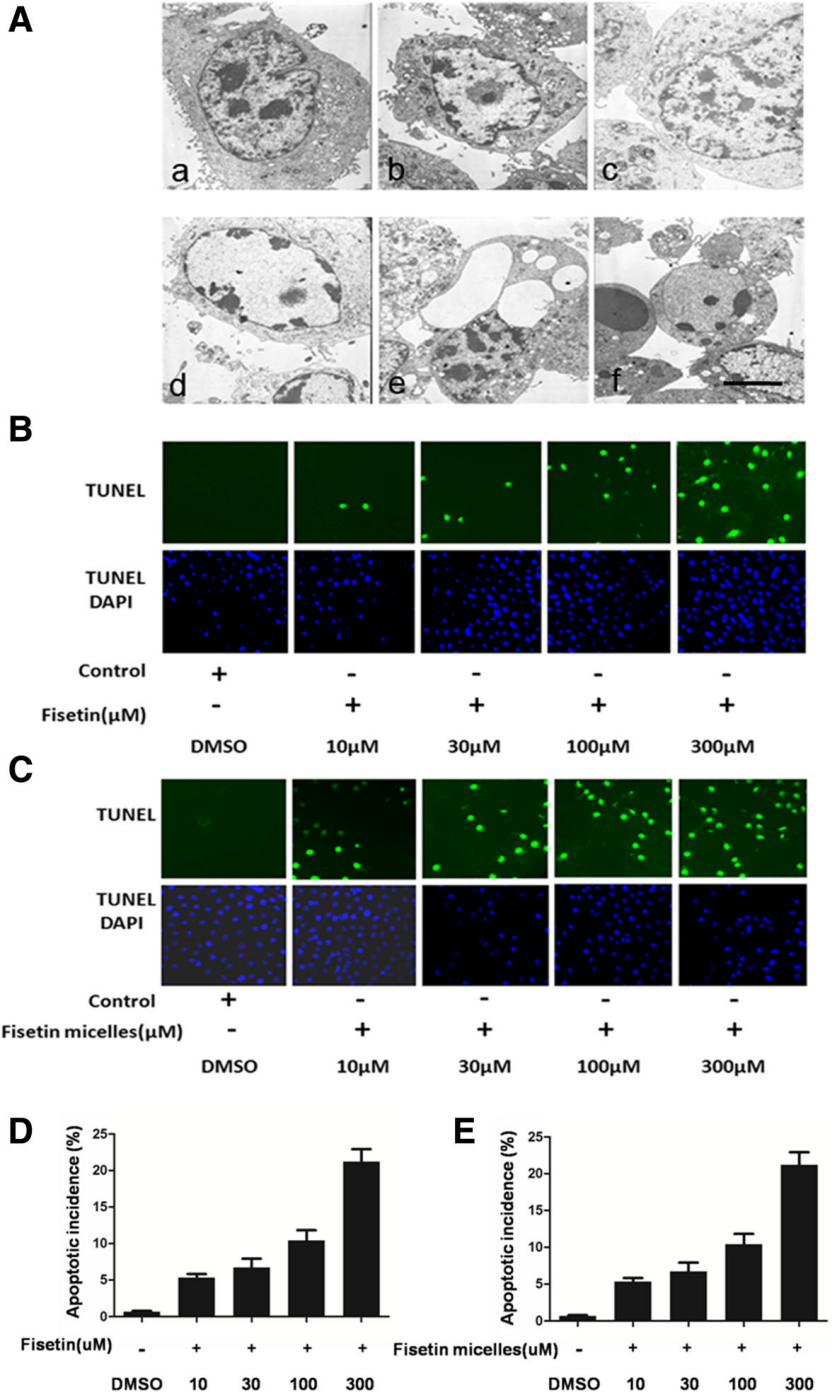
Transmission electron microscopy of SKOV3 cells treated with fisetin (300 μm) for 24 h. Images were recorded at 5000 × magnification using a transmission electron microscope. **a** (a) Normal structure of cancer cells. (b) Heterochromatin aggregation in the cell. (c) Structural changes before apoptosis. (d) Chromatin edge set and nuclear membrane thick dyeing. (e) Apoptosis cells. Membrane blebbing appeared inside the cells. (f) Apoptosis cells with apoptotic bodies, swelling of organelles. **b**-**c** SKOV3 cells were treated with different concentrations of fisetin/fisetin micelles and labeled with TUNEL and DAPI after 24 h. Images of random fields (*n* = 10) per slide (*n* = 3) were collected at 400×. **d**-**e** Quantitative analysis of the apoptosis (TUNEL) he apoptotic cells were counted with at least 100 cells from four randomly selected fields in each group. Compared with control, fisetin/fisetin micelles treated SKOV3 cells showed obvious apoptosis in concentration-dependent manner. Representation figures of treated cells labeled for TUNEL and counterstained with DAPI are shown. Scale bar,5um

To determine the apoptotic features induced by fisetin/fisetin micelles treatment, we carried out analysis using several independent approaches. Firstly, we used transmission electron microscopy to check the apoptotic bodies. Secondly, we utilized terminal deoxynucleotidyltransferase-mediated dUTP nick end labeling assay to indicate the apoptosis of fiestin/fisetin micelles-treated SKOV3 cells. Thirdly we verified our conclusion depending on western blot results. After 12 h of fisetin/fisetin micelles treatment, ovarian cancer cells shrunk, followed by cell detachment from the culture plate surface indicating anoikis. Transmission electron microscopy of fisetin/fisetin micelles-treated SKOV3 cells demonstrated ultrastructural morphological changes characteristic of apoptosis. Figure 2Ab showed the well-defined, uniform and dense chromatin under the nuclear membrane. Figure 2Ac showed changes in the kernel. The chromatin of the nucleus condensed into a heterologous particle and located in the center of the nucleus. The changes inside the cytoplasm including cytoskeletal filaments intensive, nuclear protein paritcle swarms, endopalsmic reticulum rearrangement and form concentric circle shape in cells.DNA fragmentation assay showed oligonucleosomal DNA ladders in Fisetin-treated cells (Data not shown). The results of TUNEL staining research verified that Fisetin/fisetin micelles induced apoptosis in SKOV3 cells and furthermore, showed that fisetin/fisetin micelles induced apoptosis in SKOV3 cells in a dose-dependent manner (Fig. 2).

**Fig. 3:**
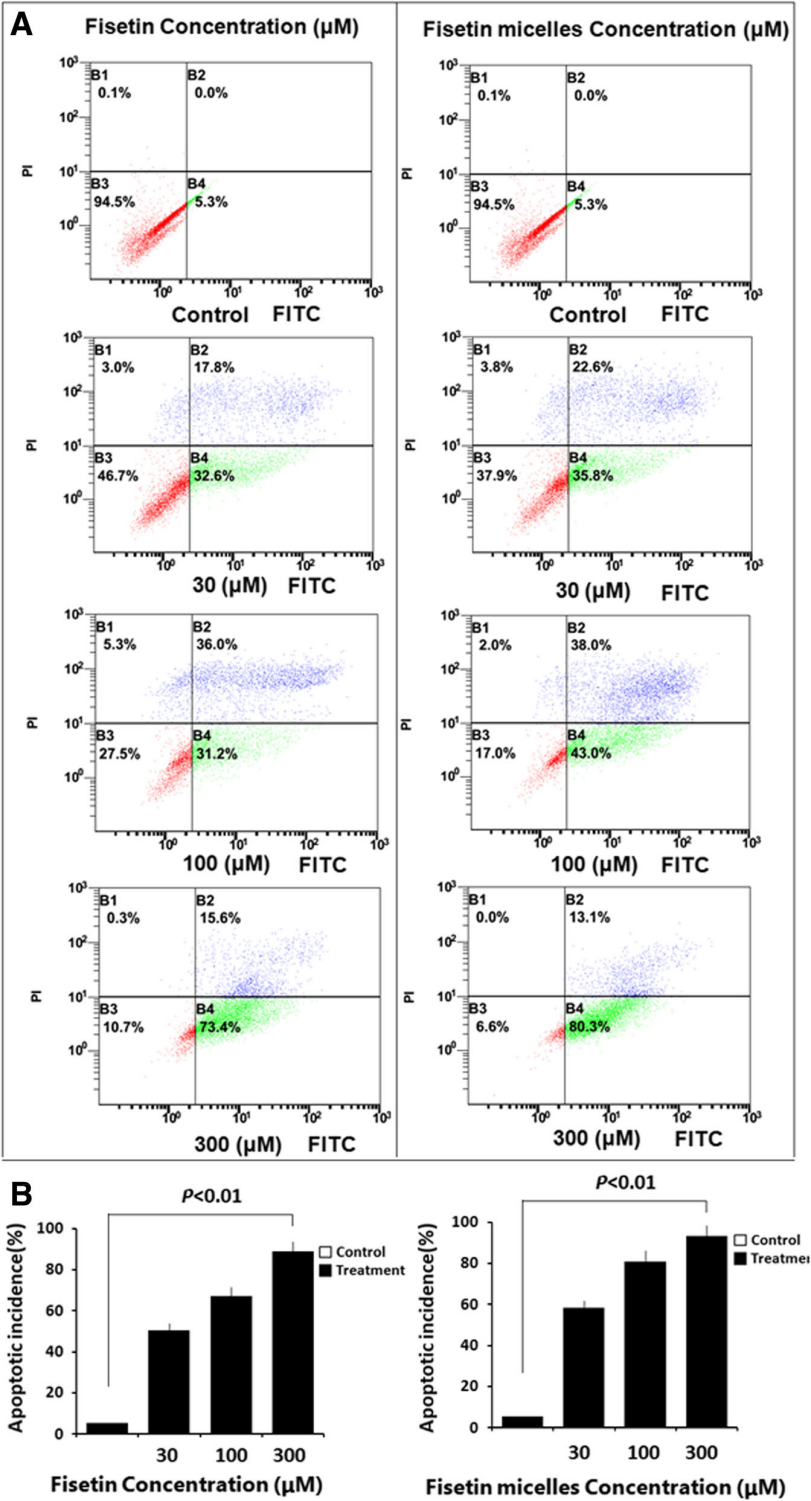
FACS analysis for apoptotic incidence. **a** SKOV3 cells were treated with different concentrations of fisetin for 24 h. SKOV3 cells were treated with different concentrations of fisetin micelles for 24 h. **b** The bar chart shows percentage of apoptotic incidence compared to untreated control. Fisetin and Fisetin micelles induced SKOV3 cell apoptosis in a concentration-dependent manner. Error bars represent standard error of the mean (SEM) (*n* = 3) (*p* < 0.01, compared to control to be considered as significant)

To quantitate apoptotic process, fluorescence activated cell sorting (FACS) analysis was carried out to detect early apoptosis marked by Annxin V and late apoptosis by PI. SKOV3 Cells treated with various concentrations (30 μM, 100 μM, 300 μM) for 24 were subjected to analysis. To further analyze cell death induced by fisetin and fisetin mieelles, the relative portions of DNA contents were assessed by flow cytometry. In Fig. 3a, the left lower quadrant of the Annexin showed alive cells (Annexin V-/PI-), and the right upper quadrant of Annexin indicated the apoptotic cells in the late stage (Annexin V+/PI+), and the right lower quadrant was the early apoptotic cells (Annexin V+/PI-). Flow cytometry annexin V/PI staining showed that the early apoptosis of SKOV3 cells treated with 300 μmol/L of festin (73.4%)at 24 h was obviously higher than cells treated with 100 μmol/L of festin(31.2%). The late-stage apoptosis of SKOV3 cells treated with 300 μmol/L of festin (15.6%)at 24 h was obviously higher than cells treated with 100 μmol/L of festin(36.0%). Concentration-dependent studies indicated an apoptosis ratio increasing compared to the control group after 24 h treatment. The similar results were shown in flow cytometry data of fisetin micelles treated SKOV3 cells. SKOV3 cells treated with multiple concentrations were analyzed. The results showed that fisetin micelles treatment induced SKOV3 cell apoptosis in a concentration-dependent manner. The apoptosis ratio is higher in fisetin-micelle treated cells, indicating a better drug kinetics (Fig. 3).

### Fisetin/fisetin micelles activate the apoptosis through mitochondrial pathway

**Fig. 4:**
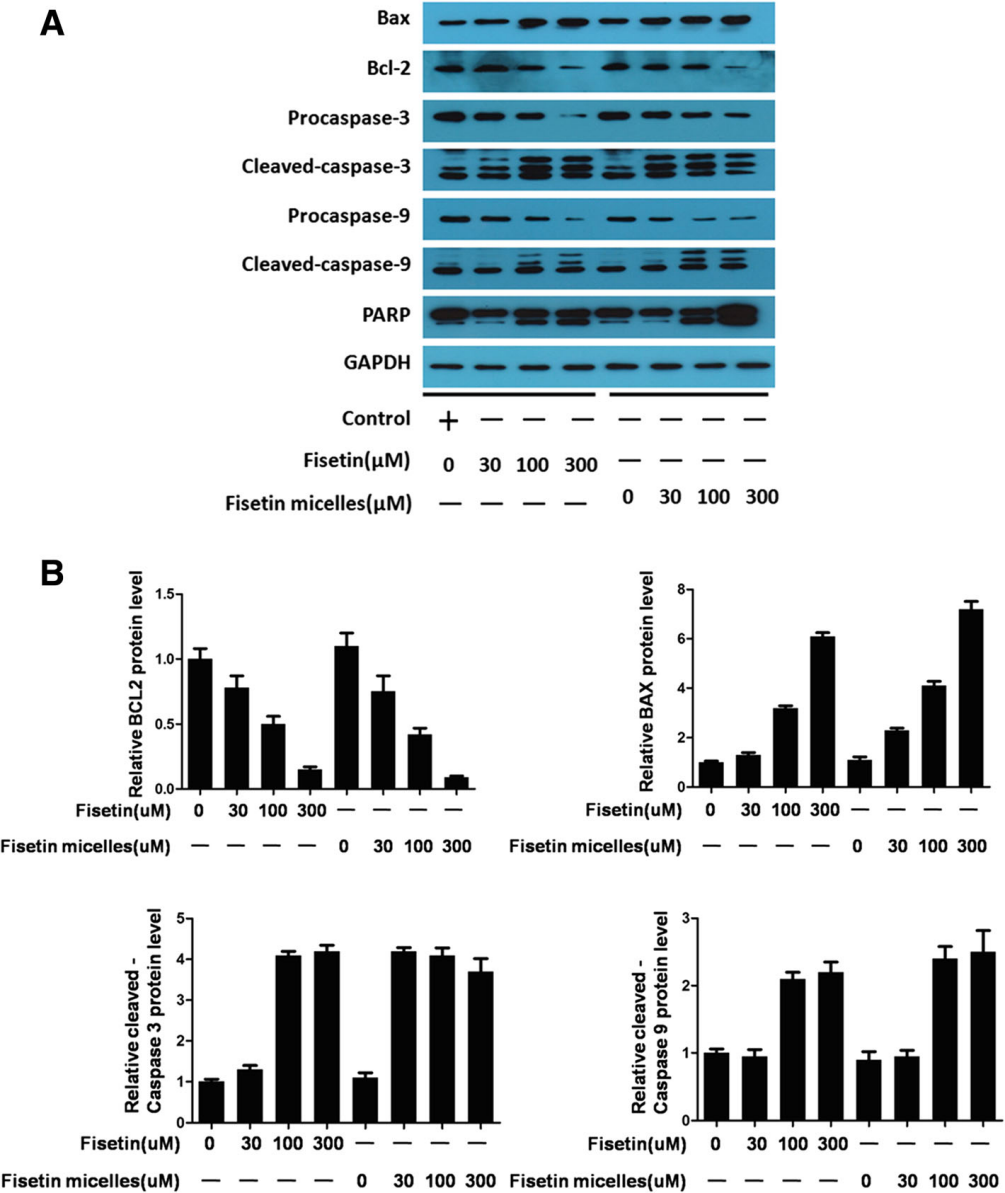
Fisetin/fisetin micelles induce cell apoptosis through mitochondrial pathway. Fisetin/fisetin micelles inducecaspase activation and imbalance of Bax/Bcl-2 in treated SKOV3 cells. **a** Cells treated with different concentrations of fisetin for 24 h, meanwhile, at the same electric lane, sample from SKOV3 cells treated with the same concentration of fisetin micelles were loaded. DMSO (< 0.1%) diluted in saline was considered as control. GAPDH was used as a loading control. **b** Densitometric analysis was performed for Bcl-2, Bax, Cleaved-caspase-9 and Cleaved-caspase-3. Values were normalized to GAPDH. *P* < 0.05 compared to control

We analyzed the sub-routes of both fisetin-induced and fisetin micelles-induced apoptosis. Fisetin activated the apoptosis by mitochondrial pathway. As shown in Fig. 4, fisetin treatment led to high expression of proapoptotic protein Bax followed by activation of Caspase-3 and Casapse-9 in a concentration-dependent manner. Pro-caspase-3 and pro-caspase-9 gradual disappeared concomitant with appearance of proteolytic cleavage bands. The downstream factors such as cytochrome *c* and PARP proteins levels were markedly increased in a concentration-dependent manner. Anti-apoptotic Bcl-2 protein levels were reduced in cells treated with fisetin at a concentration as low as 10 μM. Similarly, the imbalance of Bax/Bcl-2 appeared in SKOV3 cells treated with fisetin micelles. The results were verified by the immunochemical studies. The slides indicated fisetin/fisetin micelles broke the balance of Bax and Bcl-2. The same results were observed in the fisetin micelles-treated SKOV3 cells.

### Fisetin/fisetin micelles inhibit the tumor growth in a xenograft mouse model

**Fig. 5:**
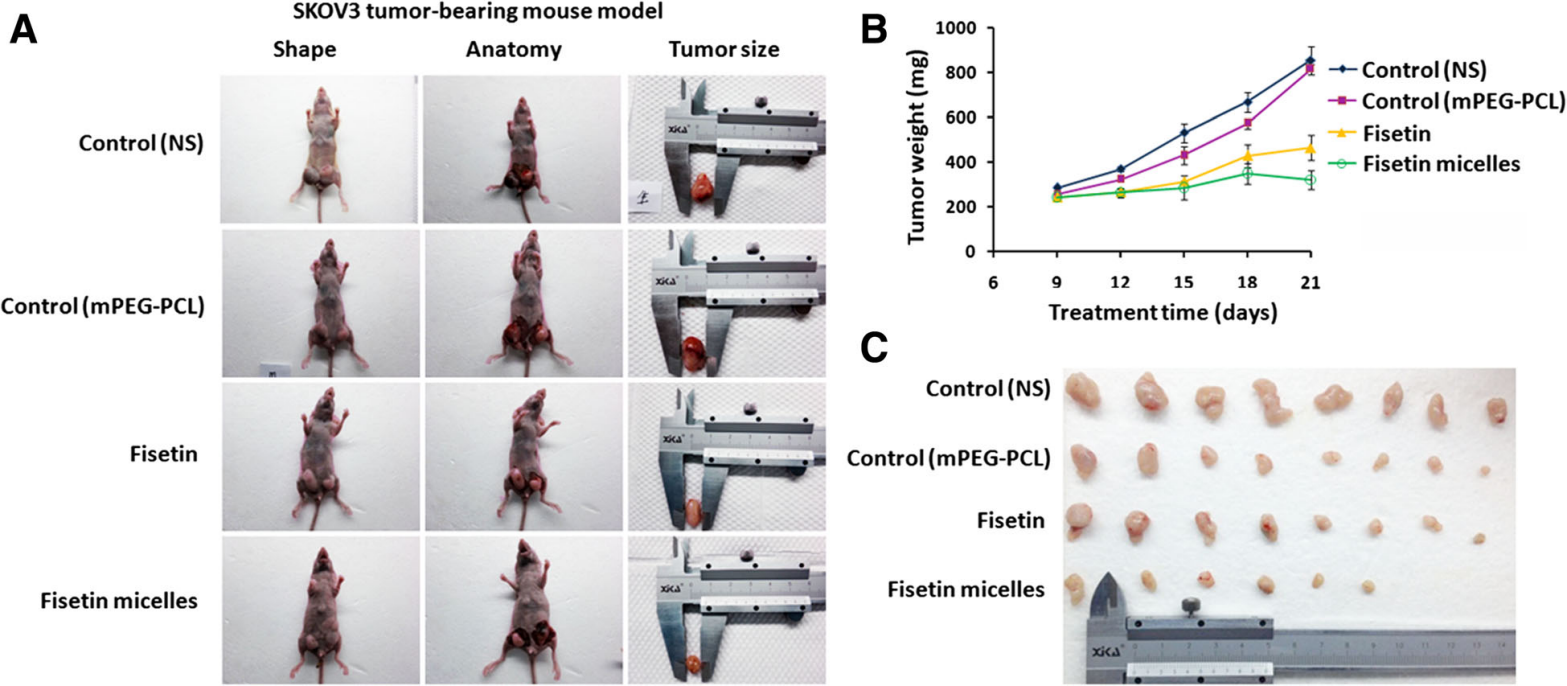
Fisetin and fisetin micelles inhibit tumor growth in a xenograft model of ovarian cancer. **a** Xenograft mice were implanted with 5 × 10^6^ SKOV3 cells on day 0 and were randomly divided into various treatment and control groups (*n* = 5). **b** Eight days after implantation, tumor-bearing mice were treated every week according to the protocols. **c** Tumor-bearing mice were treated with fisetin/fisetin micelles or received the vehicles, either DMSO or mPEG-PLC by intraperitoneal administration for 4 weeks, 4 consecutive days per week with either fisetin or fisetin micelles (50 mg/kg or 100 mg/kg). (*p* < 0.01, compared to control to be considered as significant)

The antitumor efficacy of both fisetinand fisetin micelles was determined in vivo. SKOV3 cells treated with different dose of fisetin/fisetin micelles(50 mg/kg) with DMSO and mPEG-PLC were taken as control, then were injected into well-established xenograft mouse model of ovarian cancer. Tumor growth was monitored every other day. No acute toxic effects were observed during the experiment process. Interesting, the tumor volume in fisetin-treated groups was obviously smaller than the other control groups, which treated with vehicle solution of DMSO (< 0.01%)diluted in saline solution (*p* > 0.05). Highest dose of fisetin-treated groups showed strongest tumor inhibition ability; the difference was statistically significant, which indicated that fisetin treatment significantly delayed ovarian cancer growth in dose-dependent manner. As shown in Fig. 5, fisetin micelles also indicated strong antitumor ability in xenograft mice carrying SKOV3. Most intriguing, as we have shown, although both fisetin and fisetin micelles have the same range of efficacy, fisetin micelles’ antitumor ability appeared to be marginally stronger than free fisetin. At the end of the experiment, we found that fisetin treatment at 50 mg/kg dosage led to 53.6% tumor growth inhibition. All of the data showed that fisetin can effectively decrease the tumor size and weight. The antitumor of fisetin micelles appeared to reach70.7% inhibition after 21 days of treatment. Meanwhile, at the same dose of treatment, fisetin micelles seems to be more powerful than free fisetin,

### Ultrasound scan and the expression of apoptotic factors inside the tumor tissue

**Fig. 6:**
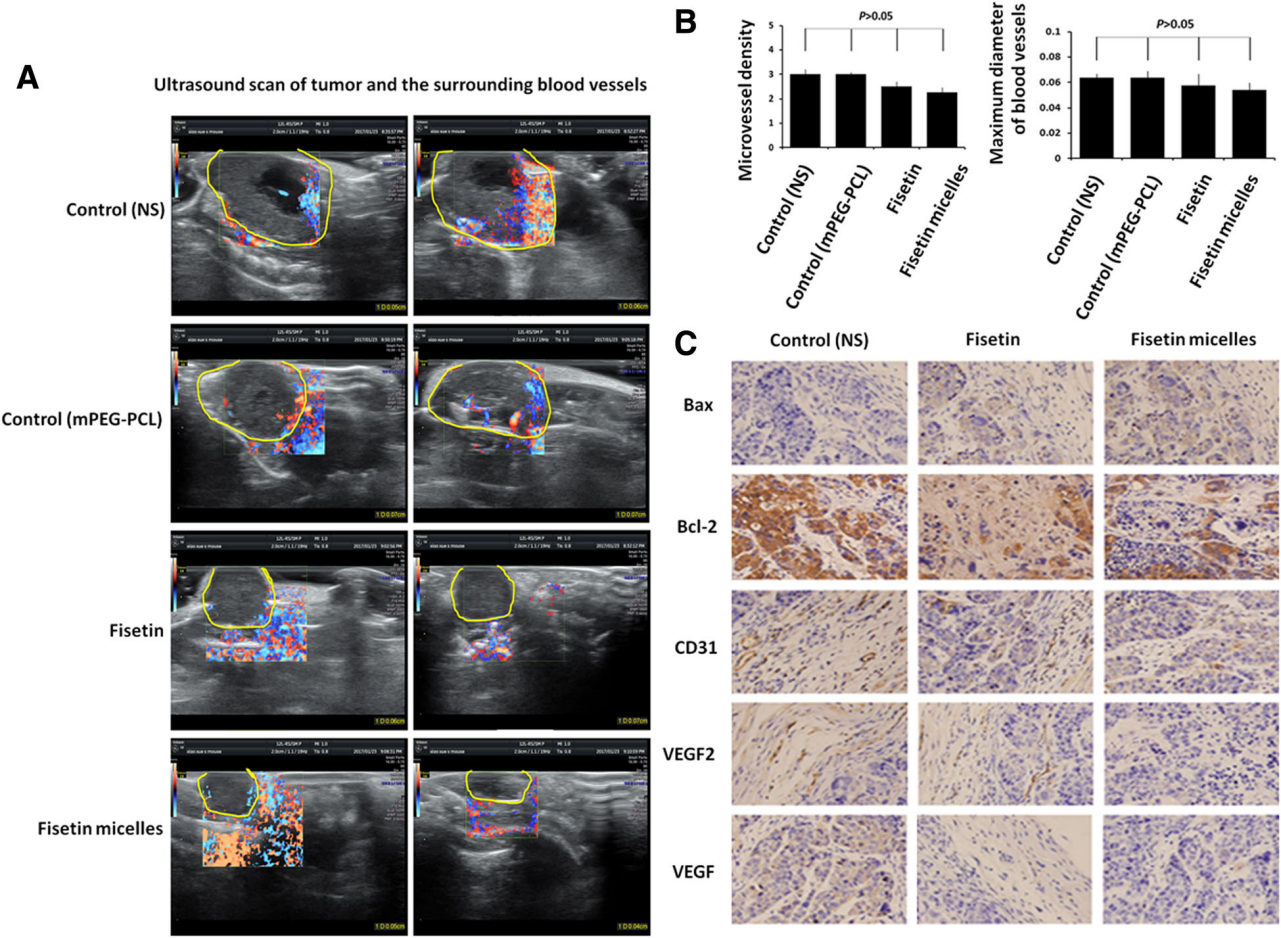
Fisetin treatment suppresses the tumor growth and tumor angiogenesis. **a** Representative images of the implanted tumors and vessels in the fisetin-treated and control mice monitored by a Philips HD11 ultrasound scanner equipped with an 11 MHz linear array transducer are shown in this figure. Yellow dotted line defined the tumor location. The red and blue color indicates flows toward and away from the ultrasound transductor of blood vessels and microvessel density deduced from the ultrasound signals from randomly chosen planes indicates that fisetin/fisetin micelles treatment affects the tumor blood vessel characteristics in SKOV3 ovarian tumor xenografts. **b** Columns, means (*n* = 3); bars SE. **c** Representative images of random sections of the grafts from fisetin/fisetin micelles treated or control mice were labeled with marker for blood vessel marker CD31 and VEGFR2 and subjected to H&E staining (images were recorded at the magnification of × 200). Figures demonstrated that fisetin/fisetin micelles treatment compromises blood vessel network and VEGFR2 expression

The volume (V) of the solid tumors was measured by a philipsHD11 ultrasound scanner (Philips Medical Systems, Best, The Netherlands equipped with an 11 MHz linear array transducer. The volume of solid tumors (expressed in millimeter) was documented in three dimensions, including length, width and height. The minimum diameter of the lesion that can be detected by ultrasound is only 0.01 cm. According to Fig. 6, the volume of tumor with treated fisetin/fisetin micelles is obviously smaller than other control groups. Meanwhile, the vessel number and size inside the tumor with fisetin/fisetin micelles treatment are less than control groups, the maximum blood vessel diameter was smaller than the untreated controls, although the statistical significance is not obvious. According to Fig. 6, at the concentration of 50 mg/kg, the blood vessel number is 2 and maximum blood diameter is 0.5 mm, the blood vessels number is less than control groups and vessel’s diameter is smaller than control.(*P* < 0.05). As we know, Bcl-2 and Bax are pair of apoptosis proteins. It is well known that cross-talk between Bcl-2 and Bax intimately links to the fate of the cell. Bax itself can form a homologous. When the proportion of Bax/Bax is more than Bcl-2/Bax, it promotes the apoptosis of cells, whereas it can inhibit the apoptosis of cells. Followed with the increasing of the concentration of fisetin/fisetin micelles, Phosphorylation of ERK was inhibited, which led to high expression of Bax and low expression of Bcl-2. In addition to activating the apoptotic pathway, fisetin/fisetin micelles also exerts its effects on ERK MARK (Data not shown). From Fig. 6c, data showed that Bcl-2 expression decreased inside the tumor tissue of xenograft treated with fisetin/fisetin micelles (*P* < 0.05), whereas Bax expression increased (*P* < 0.05), especially in the groups treated with high concentration of fisetin/fisetin micelles. The experiment indicated that Bcl-2 expression decreased and Bax expression increased in a dose-dependent manner after fesitin/fisetin micelles treatment. Fisetin/fisetin micelles treatment induced apoptosis of human ovarian cancer cells by mitochondrial pathway.

## Discussion

Ovarian cancer is the most lethiferously gynecological cancer. Among the primary malignant tumors of the ovary, the epithelial malignant tumor is the main type. In the United States, the new incidence of ovarian cancer cases is about 22440and 14080death in 2017, which is occupied 5% of the total number of death populations, accounting for fifth of all cancer deaths in the United States [1]. With the development of modern medical technology, the efficiency of surgical treatment of ovarian cancer is significantly improved. Even though, however, the outcome of ovarian cancer is not satisfactory. To improve the survival rate and quality life in patients with ovarian cancer, especially in patients with advanced ovarian cancer stage, identification of effective, safe, non-toxic and low toxicity new drugs is particularly important [23–25]. Flavonoids are widely found in all kinds of plant pigments. Previously they are widely consumed by human beings. Fisetin (3,3 ‘, 4’, 7 - 4 - hydroxy flavone) is a kind of flavonoids, which belongs to the polyphenolic compounds, extracted from the sumac plant stems and leaves of dietary. Flavonoid composition exists widely in all kinds of fruits and vegetables, such as apples, strawberries, persimmons, grapes, onions and cucumbers [26–30].

Pharmaceutical interest is increasing to discover natural compounds as well as their derivatives with anti-tumor activities [31]. In this regard, fisetin is a promising natural compound. Here we establish that it has cytotoxic effects in ovarian cancer cells by its ability to induce apoptosis and suppress angiogenesis. However, unmodified fisetin is of little clinical value due to low hydrophilicity. Attempts to increase the solubility by chemical modification always failed to maintain the anti-tumor effects. In present study, we developed fisetin micelles to improve its physical properties. Surprisingly, fisetin micelles demonstrated superior anti-tumor activities. In our study, we chose fisetin rotary evaporation with ethanol to prepare fisetin micelles due to the excellent properties of this products. The mPGE-PCL ends solve the problem of fisetin’s poor water solubility. More than that, mPEG-PCL encapsulating fisetin has small particle size, narrow distribution, high EE, high DL, good homogeneity and gradually releasing behavior. We prepared fisetin micelles according to the previous protocols. Simply, Fisetin and MPEG-PCL micelles (fisetin:MPEG-PCL/wt = 1:9) were codissolved in dehydrated ethanol, then the mixed solution was evaporated at 60 °C. Fisetin finally distributed in the MPEG-PCL copolymer and formed a homogeneous amorphous coevaporation. The self-assembly process that coevaporation dissolved in a saline was called forming fisetin micelles. According to previous drug release study of fisetin micelles, the rate of fisetin released from micelles was much slower than that of free fisetin. The sustained-release property of fisetin micelles showed 73% release rate after 6 days of observation, while the free fisetin reached 93% of release rate. The releasing delay suggested that MPEG-PCL could minimize the trauma to normal tissues and increase the accumulation in ovarian cancer tissues.

Fisetin micelles were more effective in suppressing tumor growth and prolonging survival than free fisetin. Cytotoxicity data showed that fisetin micelles had enhanced cytotoxicity compared to free fisetin (mean IC50 = 48.2 μM versus61.2 μM, *p* < 0.05). Meanwhile, fisetin micelles treated cells showed stronger apoptosis induction ability, which was verified with TUNEL assay, flow cytometry and western blot analysis. It was indicated that uptake mechanisms of fisetin micelles could be connected to some sub cellular structures such as lysosomes. What’s more, the studies in vivo further indicated that fisetin micelles had a more efficient antitumor effect through the analysis of TUNEL assay, immunohistochemical detection and western blot.

We suspect the improvement could be explained by the following reasons. Firstly, from the in vitro drug-releasing model, we found that fisetin micelles pass through the blood vessel walls toward tissues at a relatively stable speed compared to free fisetin. The drug concentration in circulation and in plasma of cells could be maintained at a satisfied level to guarantee enough drug concentration around the tumor tissues area. The limited release to normal tissues limited the body immune attack to fisetin micelles, and, mPEG-PCL micelles were safe drug carrier with low cytotoxicity to normal cells. Secondly, fisetin micelles have smaller and more homogeneity size making transfer easier. Different from normal tissues, blood vessels inside tumor tissues have leaky vascular architecture, which means a big gap between endothelial cells as large as 20-2000 nm. The gap seems easy for fisetin micelles to penetrate while might be a barrier for free fisetin.

## Conclusion

Biodegradable fisetin micelles have been successfully structured and applied to the SKOV3 xenograft tumor model. Compared to free fisetin, fisetin micelles of small particle size and good homogeneity demonstrated lasting in vitro release behavior, cellular uptake, and enhanced cytotoxicity, as well as improved apoptosis induction. Fisetin micelles were considered to be potential antitumor drug after improvement of its solubility and bioavailability.
